# Is speciation driven by cycles of mixing and isolation?

**DOI:** 10.1093/nsr/nwy113

**Published:** 2018-10-08

**Authors:** Nicholas H Barton

**Affiliations:** 1 Institute of Science and Technology Austria; 2 Austria Reviewer of NSR

He *et al*. [1] describe genetic divergence between mangrove species that are distributed across the Straits of Malacca. Divergence varies much more across the genome than in a simple null model of strict allopatry. Such heterogeneity (often termed ‘genomic islands of speciation’) can be generated by population structure: populations that exchange genes at a low rate will show wide variability in divergence, depending on when migration events occurred on the ancestral lineages that trace back from each section of genome. He *et al*. [1] also used the pairwise sequentially Markov coalescent to estimate that the species’ effective population size increases back into the past. Again, this can be explained by population subdivision: lineages sampled from the same place either quickly coalesce, or wander across the species’ range as they trace back into the past, reducing the rate of coalescence, and therefore increasing the species-wide effective size. These observations are consistent with intermittent isolation and mixing caused by changes in sea level. However, as He *et al*. [1] acknowledge, they may be produced by many other processes: for example, selection or variation in recombination rate [2]. Thus, the best evidence for intermittent mixing is geological.

In their abstract, He *et al*. [1] argue that ‘speciation… is driven by cycles of isolation and gene flow’, and that ‘the mixing–isolation–mixing (MIM) mechanism of speciation is… efficient, potentially yielding mn (m>1) species after n cycles’. Both claims can be contested.

First, there is no evidence as to the causes of speciation. In this mangrove example, we have good geological evidence for intermittent contact and we see high species diversity. However, to show a connection between the two, one would need to establish that the actual reproductive isolation and ecological divergence were caused (or at least facilitated) by intermittent gene flow. This is extremely challenging. Alternatively, a comparative study might show a significant association between intermittent geographic barriers and species diversity. This seems more feasible, but nevertheless would require very many replicate species groups.

Second, He *et al*. [1] argue that MIM gives an efficient mechanism of speciation, because each cycle multiplies the number of species by some factor, leading to an exponential growth in species numbers. Yet, any speciation mechanism can potentially lead to exponential growth if one assumes that each species can split into multiple species at some rate (of course, in reality, species numbers are limited by available locations and niches). More fundamentally, this argument takes ‘speciation’ to be a discrete event. In fact, it is a process by which multiple differences evolve independently, and then gradually come together to give distinct and incompatible populations that we recognize as species [3]. Multiple episodes of secondary contact (i.e. of MIM) or, less drastically, expansions and contractions of local populations, can bring together differences that evolved independently, producing hybrid zones and ultimately full species [4]. This process may play an important role in speciation; yet, even with an abundance of genomic data, it is hard to see how to estimate its importance.
